# Arsenic 3 methyltransferase (AS3MT) automethylates on cysteine residues in vitro

**DOI:** 10.1007/s00204-022-03248-8

**Published:** 2022-03-04

**Authors:** Sofiane Y. Mersaoui, Cynthia Guilbert, Hsiang Chou, Christelle Douillet, D. Scott Bohle, Miroslav Stýblo, Stéphane Richard, Koren K. Mann

**Affiliations:** 1 Segal Cancer Center, Lady Davis Institute for Medical Research and Departments of Oncology and Medicine, McGill University, Montréal, Québec H3T 1E2 Canada; 2 Department of Nutrition, Gillings School of Global Public Health, University of North Carolina at Chapel Hill, CB# 7461, Chapel Hill, NC 27599 USA; 3 Department of Chemistry, McGill University, Otto Maass 233A, Montréal, Québec H3A 0B8 Canada

**Keywords:** Arsenic, AS3MT, Methyltransferase, Automethylation, Cysteines

## Abstract

Arsenic toxicity is a global concern to human health causing increased incidences of cancer, bronchopulmonary, and cardiovascular diseases. In human and mouse, inorganic arsenic (iAs) is metabolized in a series of methylation steps catalyzed by arsenic (3) methyltransferase (AS3MT), forming methylated arsenite (MAsIII), dimethylarsenite (DMAIII) and the volatile trimethylarsine (TMA). The methylation of arsenic is coordinated by four conserved cysteines proposed to participate in catalysis, namely C33, C62, C157, and C207 in mouse AS3MT. The current model consists of AS3MT methylating iAs in the presence of the cofactor S-adenosyl-L-methionine (SAM), and the formation of intramolecular disulfide bonds following the reduction of MAsV to MAsIII. In the presence of endogenous reductants, these disulfide bonds are reduced, the enzyme re-generates, and the second round of methylation ensues. Using in vitro methylation assays, we find that AS3MT undergoes an initial automethylation step in the absence of iAs. This automethylation is enhanced by glutathione (GSH) and dithiothreitol (DTT), suggesting that reduced cysteines accept methyl groups from SAM to form S-methylcysteines. Following the addition of iAs, automethylation of AS3MT is decreased. Furthermore, using a Flag-AS3MT immunoprecipitation coupled to MS/MS, we identify both C33 and C62 as acceptors of the methyl group in vivo. Site-directed mutagenesis (C to A) revealed that three of the previously described cysteines were required for AS3MT automethylation. In vitro experiments show that automethylated AS3MT can methylate iAs in the presence of SAM. Thus, we propose that automethylated may represent an active conformation of AS3MT.

**Supplementary Information:**

The online version contains supplementary material available at 10.1007/s00204-022-03248-8.

## Introduction

**Fig. 1:**
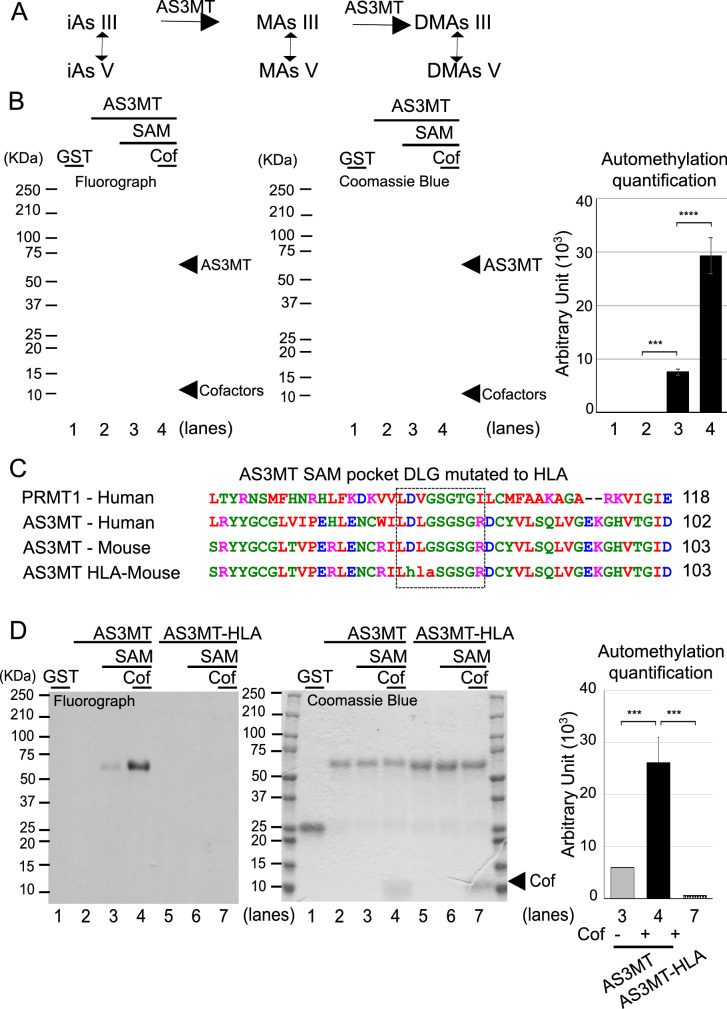
Automethylation of AS3MT requires the SAM-binding pocket. **A** Schematic of arsenic methylation. Inorganic arsenic (iAs) is methylated to methylarsenic (MAs) and subsequently to dimethylarsenic (DMAs) via AS3MT. **B** Representative blot of the in vitro methylation assay (left panel), Coomassie Blue (middle panel) and corresponding quantification signal (arbitrary unit) obtained by fluorograph (right panel) of GST or GST-AS3MT, in the presence of indicated factors (0.4 μM SAM and cofactors: 0.2 μM TRR, 10 μM TRX, 300 μM NADPH, 1 mM GSH) after a 16 h incubation at 37 °C. **C** Multiple-sequence alignment of hPRMT1 and AS3MT mouse and human showing conservative region (open box) of the SAM pocket. Three conserved residues (DLG) were mutated to (HLA) in the mutant AS3MT-HLA. **D** Representative in vitro methylation assay (left panel), Coomassie Blue (middle panel) and corresponding quantification signal (arbitrary unit) obtained by fluorograph (right panel) of the AS3MT and the AS3MT methyltransferase-dead mutant (AS3MT-HLA). Cof represents cofactors. Error bars represents standard error of the mean (SEM) from three independent experiments (*n* = 3). *** indicates *P* < 0.001, and **** *P* < 0.0001

Arsenic is an important environmental contaminant to which millions of people are exposed worldwide. Inorganic arsenic (iAs) is biotransformed (metabolized) through a series of methylation reactions catalyzed mainly by arsenic (3) methyltransferase (AS3MT), an enzyme conserved from bacteria to man (Kubota et al. 2002). The only known function of AS3MT is to methylate arsenic. AS3MT^−/−^ mice are viable with no overt phenotype, but are severely impaired in their ability to methylate arsenic (Drobna et al. 2009). The most recently proposed pathway of arsenic methylation converts iAs III to methylated arsenite (MAs III), and dimethylarsenite (DMA III), and in some species, to trimethylarsine (TMA) (Fig. 1A), with oxidation reactions generating methylated arsenate (MAs V) and dimethylarsenate (DMA V) (Styblo et al. 2021). S-adenosyl-L-methionine (SAM) acts as the methyl donor and at least in vitro, other cofactors, such as glutathione (GSH), thioredoxin (TRX), and thioredoxin reductase (TRR), act to support the methylation reaction (Dheeman et al. 2014; Thomas et al. 2004).

Importantly, single nucleotide polymorphisms (SNPs) in the *AS3MT* gene have been shown to affect its methyltransferase activity (Drobna et al. 2009; Li et al. 2017). The importance of these SNPs, and iAs methylation in general, to toxic outcomes is poorly understood. It was suggested, however, that certain SNPs are associated with a higher risk of disease including skin lesions (Wu et al. 2014), atherosclerosis, diabetes (Wu et al. 2014), and cancer (Beebe-Dimmer et al. 2012; Engstrom et al. 2015). Historically, biotransformation of arsenic was thought to be a “detoxification” process leading to a more readily excretable compound. However, some methylated intermediates, particularly MAs III, can be more cytotoxic than iAs (Petrick et al. 2000; Styblo et al. 2000; Sumi and Himeno 2012; Yoshinaga-Sakurai et al. 2020). Mice deficient in AS3MT are protected against arsenic-induced atherosclerosis, suggesting arsenic methylation may contribute to some of these outcomes (Negro Silva et al. 2017). Thus, factors that modulate AS3MT enzymatic efficiency may be important variables in arsenic-associated toxicities.

AS3MT is linked to pathological changes in the absence of arsenic. Genome-wide association studies link genetic variation in *AS3MT* (chromosome 10q24) with schizophrenia (Duarte et al. 2016) and ischemic heart disease (Winsvold et al. 2017). *AS3MT* null mice are associated with sex-specific metabolomic changes (Huang et al. 2017, 2016). *AS3MT*^*−*/−^ mice have a greater body mass with a higher percentage of adipose tissue (Douillet et al. 2017). Although the relevance of this is unclear, many of the consequences of iAs exposure are linked to an imbalance in iAs metabolism, such as diabetes and cardiovascular diseases (Drobna et al. 2009; Moon et al. 2018).

AS3MT belongs to the superfamily of seven-β-strand methyltransferases (MTases) formed by six parallel β-strands, with the seventh anti-parallel inserted between the fifth and sixth β-strands. MTases methylate a wide range of substrates, such as lipids, proteins, nucleic acids, and small molecules (Kagan and Clarke 1994). AS3MT was first purified from rat liver, which permitted cloning of the corresponding gene (Lin et al. 2002; Walton et al. 2003). Various orthologous genes were then identified coding for proteins ranging from 348 to 382 residues with three conserved sequence motifs (Thomas et al. 2007). Interactions between SAM and these conserved amino acid motifs are critical for methyl group transfer to arsenic (Kagan and Clarke 1994; Kozbial and Mushegian 2005). SAM has an activated methyl group that is transferred by AS3MT to acceptor groups involving a nucleophile attack on the methyl group of the SAM and concomitant release of the reduced S-adenosyl-homocysteine (SAH). AS3MT also contains multiple cysteine residues, of which five are fully conserved and essential for arsenic methylation (Thomas et al. 2007). Based on the 375 residues sequence of human AS3MT, these cysteines are in positions C32, C61, C85, C156, and C206, corresponding to the positions C33, C62, C86, C157, and C207 of the mouse AS3MT protein, which contains 376 amino acids. (Thomas et al. 2007). Although many MTases have been described to be active in the dimeric form, AS3MT is a monomer in solution (Packianathan et al. 2018). Several models have been proposed for iAs methylation (Dheeman et al. 2014; Hayakawa et al. 2005). The SAM-methyl group is transferred directly to arsenic clamped by cysteines in four steps (Dheeman et al. 2014; Marapakala et al. 2012; Packianathan et al. 2018). The trivalent iAs III binds initially to the three conserved cysteine residues C62, C157 and C207. In the meantime, the methyl group of the SAM is transferred to trivalent As (III) oxidizing it to positively charged pentavalent MAs (V) species that remains bound to C33, C157 and C207. A disulfide bond forms between C33 and C62 after electrons from C62 reduce MAsV to MAsIII. The disulfide bond is reduced by thioredoxin, regenerating the active form of the enzyme, which can then methylate MAsIII to form DMAsIII (Packianathan et al. 2018).

Herein, we identify AS3MT to contain S-methylcysteines at C33 and C62 in the presence of its cofactors. We used in vitro methylation assays and immunoprecipitation-coupled mass spectrometry analyses and identified mouse and human AS3MT to be both stably automethylated on cysteine residues.

## Materials and methods

### Reagents

Arsenicals, iAs (Sigma S7400, NaAsO_2_, purity equal or more than 90%) prepared as a 10 mM stock in ddH_2_O was further diluted in H_2_O to the desired concentrations. Methyl arsonous acid (MAs III; 0.37 mg/L) synthesis was performed as previously described (Negro Silva et al. 2021) and its stock concentration was 25 mM. DMA (Sigma C4945, Sodium cacodylate trihydrate, (CH_3_)_2_AsO_2_Na 3H_2_O) stock was 10 mM in ddH_2_O. Rat liver cofactors thioredoxin (TRX, Sigma T0910) and thioredoxin reductase were purchased from Sigma (TRR, Sigma T9698), and L-glutathione reduced (GSH, Sigma G6529), NADPH (Sigma 10107824001), DTT, Adenosyl-L-Methionine (SAM), and S-[methyl-^3^H] (SAM [^3^H], PerkinElmer). Human recombinant histones H2A, H2B, H3, and H4 were purchased from New England Biolabs.

### Flag constructs

The original plasmid with the mouse AS3MT cDNA was purchased from Harvard Medical School Plasmid (clone: MmCD003315546). mAS3MT was amplified by PCR using the primers in Table 1. PCR product was digested with *Not*I and *Xba*I and subcloned in the same restriction sites in p3xFlag-CMV-14 (Sigma) plasmid. Mutagenesis was performed using Phusion Hot Start II DNA Polymerase (Thermo Fisher) with 2 h *Dpn*I (New England Biolabs) digestion step after PCR. All the mutants were created using primers in Table 1. *E. coli* DH5α strain (Civic Bioscience) was used. DNA sequencing was performed at every step of cloning to ensure accuracy of plasmids.

**Table 1:** List of primers used for the cloning of mAS3MT into p3xFlag-CMV-14, for the cloning of mAS3MT in pGEX-6P-1 and for the generation of the different mutants.

Cloning	Forward primer	Reverse primer
mAS3MT in p3xFlag-CMV-14	GGCCGCGGCCGCGACGTGGAGATCGTGAGTCATGGCTG	GGCCTCTAGAGCAGTTTTTCCTCTTGCCACAGCAGCC
mAS3MT in pGEX-6P-1	GCGCCGGCGTCGACTGGCTGCTTCCCGAGACGCTGATG	GCCGCGGCGCGGCCGCCTAGCAGTTTTTCCTCTTGCCACAGCAGCC
Mutation	Forward primer	Reverse primer
DLG to HLA	GGAAAACTGCCGAATTTTGCATCTGGCTAGTGGGAGTG	GCAATCCCTGCCACTCCCACTAGCCAGATGCAAAATTCG
C33A	GACCTCCAGACTAATGCTGCTGTCACG	CCGGCTTGGCTCGCGTGAGCCAAGCAT
C62A	GTTCGAGGTATTATGGCGCTGGTCTGACTG	GGAACAGTCAGACCAGCGCCATAATA
C157A	GCTATGATATTGTCATATCCAACGCTGTTATCAACCTTG	GTTTATCAGGAACAAGGTTGATAACAGCGTTGGATATG
C157S	GCTATGATATTGTCATATCCAACAGTGTTATCAACCTTG	GTTTATCAGGAACAAGGTTGATAACACTGTTGGATATG
C207A	GCACAAAGTTTTATGGGGGGAAGCCCTGGGAGGCGC	GATCCTTCCAGTACAGAGCGCCTCCCAGGGCTTCCC
C207S	GCACAAAGTTTTATGGGGGGAAAGCCTGGGAGGCGC	GATCCTTCCAGTACAGAGCGCCTCCCAGGCTTTCCC

### GST-mAS3MT and MBP-hAS3MT constructs

DNA fragments of mAS3MT were amplified from p3Xflag-CMV-14-mAS3MT (previously engineered construct) using primers in Table 1. The PCR product was digested with *Sal*I and *Not*I (New England Biolabs), then inserted in pGEX-6P-1 (Sigma) vector. Mutants were created with the same primers as Flag constructs (see Table 1). Protein production and purification was performed with Pierce Glutathione Agarose (Thermo Fisher) according to the manufacturer’s instructions. Briefly, 250 mL of culture was grown to O.D. of 0.6, then 0.1 mM IPTG (Sigma) induction was performed at 16 °C for 15 h. Cells were lysed, on ice for 30 min, with 10 mL of buffer (20 mM Tris pH 8.0, 150 mM NaCl, 1 mM EDTA) with protease inhibitor cocktail (cOmplete Mini, Sigma) and 1 mg/mL lysozyme (Bio Basic). Five rounds of sonication at 45% amplitude followed by incubation of 30 min on ice with the addition of Triton-X-100 (1% final concentration, Sigma) and DNase I (10 μg/mL, Bio Basic). Supernatant was cleared with a 30 min centrifugation at 20, 000 rpm. 150 μL of slurry beads was used per pull-down. Purity and relative concentration of proteins were confirmed by gel electrophoresis followed by Coomassie Blue staining.

The plasmids expressing MBP-hAS3MT were provided by Barry Rosen Lab (Dheeman et al. 2014). Briefly, the cultures were grown to reach O.D. of 0.6, induced for 4 h with 0.3 mM IPTG, then cells were pelleted and frozen at − 80 °C. The next day, cells were lysed with the buffer (50 mM MOPS pH 7.4, 10% glycerol, 0.3 M NaCl) containing protease inhibitor cocktail and lysozyme (see concentration above). Sonication and DNase I steps are the same as previously described. Purification was conducted according to the manufacturer’s protocol (Amylose Magnetic Beads, New England Biolabs). Elution was achieved with buffer 50 mM MOPS pH 7.4, 10% glycerol, 0.3 M NaCl containing fresh 10 mM maltose.

### In vitro methylation assay

In vitro methylation assay was performed as described previously (Mersaoui et al. 2019). Briefly, reaction mixtures contained 100 mM Tris–HCl buffer (pH 7, 4), recombinant protein (5 μg), 0.4 μM radiolabeled SAM [^3^H] (15 Ci/mmol stock solution, 0.4 μM final concentration, PerkinElmer), 0.2 μM TRR, 10 μM TRX, 300 μM NADPH, 1 mM GSH and with or without iAs (0.1, 1 or 10 μM). The order of addition to the reaction mixture was as follows: AS3MT protein in Tris buffer, cofactors (TRX, TRR, NADPH then GSH), arsenicals at the specified concentration when indicated, and finally, the radioactive SAM. The reaction was incubated at 37 °C during 16 h. Reaction was stopped by adding Laemmli buffer, samples were separated by SDS-PAGE and the gel stained with Coomassie Blue. After de‐staining, the gel was incubated for 1 h in EN^3^HANCE (PerkinElmer) followed by 30 min wash in cold water, according to the manufacturer’s instructions and the reaction was visualized by fluorography.

### Dialysis and secondary methylation assay

After the initial in vitro methylation as described above, samples were dialyzed using a Slide-A-Lyzer Mini Dialysis Device, 20 K MWCO (ThermoFisher, 69590). SAH (0.8 nM) was added to each sample, prior to loading in the dialysis chamber. Slide-A-Lyzers were immersed in PBS with 0.8 nM SAH and stirred gently for 6 h. Samples were transferred to Amicon Ultra-0.5 Centrifugal Filter Units (Millipore, UFC503024), washed 3 times with PBS and concentrated to 10 µL per reaction of secondary methylation (as per manufacturer’s instructions). Secondary methylation assay was performed as described above.

### HG-CT-AAS analysis of products of in vitro reaction

Speciation analysis of As was carried out directly in the assay mixtures. Samples sent to analysis were incubated with 1 μM iAs, unless stated otherwise. Aliquots of the mixtures were treated with L-cysteine to reduce pentavalent As species to their trivalent counterparts and analyzed by hydride generation atomic absorption spectrometry coupled with a cryotrap as previously described (Currier et al. 2011). This analysis determined concentrations of iAs, MAs and DMA. Total As concentration was calculated as sum of iAs, MAs, and DMA. The instrumental limits of detection for iAs, MAs and DMA using this method are 14, 8, and 20 pg As, respectively (Hernández-Zavala et al. 2008). Trimethylated As metabolites were not detected in the methylation assays with or without treatment with L-cysteine, which is consistent with previous studies using human AS3MT (Ding et al. 2012).

### Mass spectrometry in AML12 cells

AML12 cells (ATCC) were grown in DMEM/F12 (Wisent) supplemented with 10% FBS (Wisent), insulin (Sigma), Holo-Transferrin (Sigma), Dexamethasone (Sigma). Cells were stably transfected with either the empty vector (EV) p3X-flag or the p3X-WTmAS3MT-flag constructs and selected with 900 μg/mL of G418 (Wisent). Immunoprecipitation using Flag M2 beads (Sigma) was performed according to manufacturer’s instructions. Beads were washed and sent to MS/MS for analysis at Université de Sherbrooke. The analysis was performed as previously described (Dubois et al. 2016).

### Murine AS3MT model generation

The AS3MT model was generated using the fully automated Swiss Model website (https://swissmodel.expasy.org/) and the FASTA protein sequence of AS3MT from mus musculus (NM_020577.3). The website automatically matches sequence based on its pre-existing database. This particular sequence was constructed based on 6CX6. Using the site’s available tools, pictures were taken to show the individual cysteine molecules of interest. Purple = arsenic, yellow = sulfur, red = oxygen, blue = nitrogen, grey = carbon.

## Results

### Automethylation of AS3MT requires the SAM-binding pocket

In addition to its well-known substrate arsenic, we examined whether AS3MT had protein methylation activity towards exogenous substrates. Histones are well-characterized targets of methyltransferases (Chi et al. 2010; Guccione and Richard 2019). We performed an in vitro protein methylation assay using recombinant mouse AS3MT glutathione S-transferase (GST) fusion protein purified from bacteria and a radiolabeled S-adenosyl-L-methionine (SAM) [^3^H] to visualize the methylation of potential substrates (Bedford et al. 2000). After 16 h, the reaction was analyzed by SDS-PAGE followed by fluorography. AS3MT was unable to methylate free histone H2A, H2B, H3, or H4 (Fig. S1), even in the presence of cofactors known to facilitate arsenic methylation, namely GSH, NADPH, TRR and TRX (Aposhian et al. 2003; Ding et al. 2012; Mandal et al. 2001; Thomas et al. 2004); however, we did observe the automethylation of AS3MT (Fig. S1). Automethylation of GST-AS3MT was weakly observed without cofactors (Fig. 1B, lane 3) and it was significantly (threefold) enhanced with cofactors (Fig. 1B, lane 4).

SAM is known to contact nine key residues (LDLGSGSGR; Fig. 1C) forming the SAM-binding pocket (Martin and McMillan 2002; Petrossian and Clarke 2009). To show that the AS3MT methyltransferase activity was required for the automethylation, we substituted DLG to HLA within the SAM-binding pocket (Fig. 1C) to generate an AS3MT methyltransferase-dead mutant (AS3MT-HLA). As expected, AS3MT-HLA was unable to automethylate itself (Fig. 1D), suggesting the methyltransferase activity of AS3MT was required for the observed methylation events. Furthermore, the observed automethylation of the AS3MT was not limited to mouse AS3MT, as human recombinant MBP (maltose binding protein)-AS3MT fusion protein, was also detected in an in vitro methylation assay (Fig. S2). These finding suggest that automethylation of mouse and human AS3MT is a conserved event (Fig. S2).

### Arsenic substrates compete with the automethylation

**Fig. 2:**
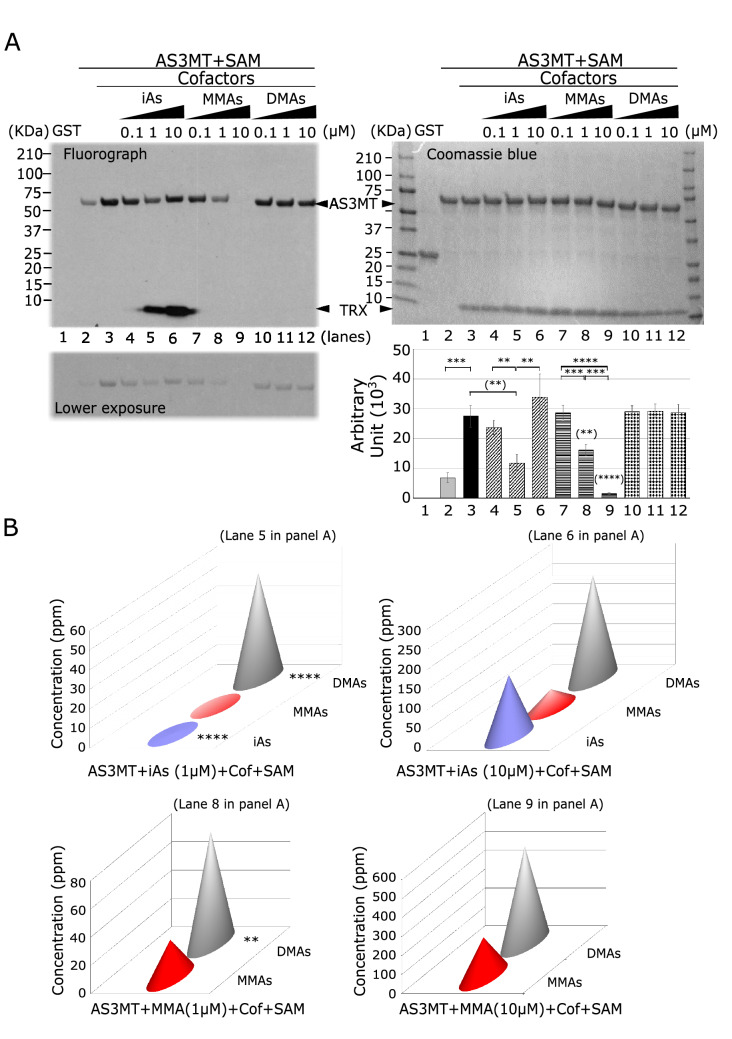
Arsenic substrates compete with the automethylation. **A** Representative in vitro methylation assay during 16 h in the presence of various arsenicals (iAS, MAs or DMA) with increasing amount (0.1 µM, 1 µM and 10 µM) as indicated in top left panel. The bottom left panel is the same with lower exposure to confirm lack of overexposure. Top right panel is the Coomassie Blue for loading control, and bottom right panel corresponding to the quantification signal (arbitrary unit) obtained by fluorograph of AS3MT automethylation. Error bars represents standard error of the mean (SEM) from three independent experiments (*n* = 3). ** indicates *P* < 0.01, *** *P* < 0.001, and **** *P* < 0.0001. When indicated between () is compare to lane 3. **B** Elution profile of arsenicals obtained from HPLC/ ICP-MS corresponding to the in vitro methylation assay from panel A as indicated. Amount of arsenicals (iAs, MAs and DMA) are expressed in ppm. Error bars represents standard error of the mean (SEM) from two independent experiments (*n* = 2). *** indicates *P* < 0.001 when compared to the control condition (AS3MT + iAs + SAM)

To test the effect of arsenic addition on AS3MT automethylation, we performed the in vitro methylation assay reaction in the presence of various arsenicals (iAsIII, MAsIII or DMAV) with increasing concentration (0.1 µM, 1 µM or 10 µM). The AS3MT automethylation decreased with iAs at 0.1 and 1 µM but not 10 µM (Fig. 2A, lanes 4–6), while MAsIII resulted in a dramatic decrease of the automethylation in a concentration dependent manner (Fig. 2A, lanes 7–9). DMAV had no effect on AS3MT automethylation (Fig. 2A, lanes 10–12). Of note, a faster migrating methylated protein with the molecular mass of TRX was observed in an iAs dose-dependent manner (Fig. 2A, lanes 4–6). We performed arsenic methylation experiments using the in vitro assays and hydride-generation atomic absorption spectrometry coupled with a cryotrap (HG-CT-AAS) to confirm GST-AS3MT was methylating arsenic. Both iAsIII and MAsIII were methylated by GST-AS3MT under these conditions (Fig. 2B). These findings show AS3MT automethylation is competing with iAsIII and MAsIII substrates.

### Reducing conditions facilitate AS3MT automethylation

**Fig. 3:**
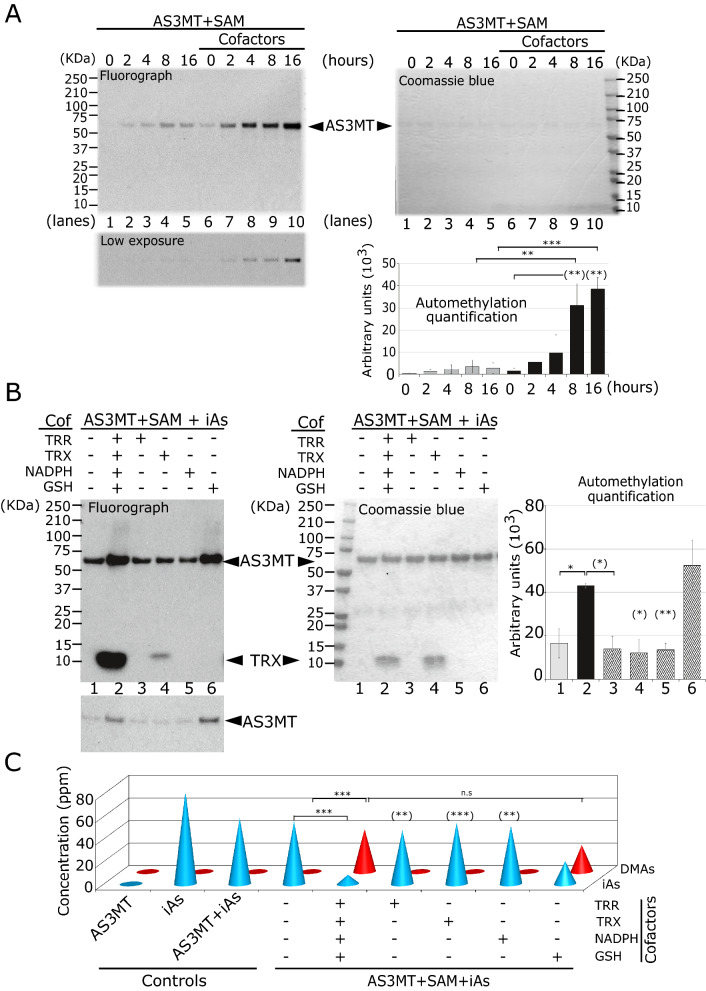
GSH is sufficient for AS3MT automethylation. **A** Representative data from a time course of the in vitro methylation assay over 16 h in the presence or absence of cofactors as indicated in top left panel. The bottom left panel is the same with low exposure to confirm lack of overexposure. Top right panel is the Coomassie Blue for loading control, and bottom right panel corresponding to the quantification signal (arbitrary unit) obtained by fluorograph of AS3MT automethylation. **B** Representative in vitro methylation assay (left panel), Coomassie Blue (middle panel) and quantification signal (arbitrary unit) obtained by the fluorograph (right panel) of AS3MT in the presence of indicated cofactors 0.2 μM TRR, 10 μM TRX, 300 μM NADPH, 1 mM GSH and 1 μM iAs. **A**, **B** Error bars represents standard error of the mean (SEM) from three independent experiments (*n* = 3). * indicates *P* < 0.05, ** indicates *P* < 0.01, *** *P* < 0.001, and **** *P* < 0.0001. **C** Arsenic speciation profile obtained from HPLC/ICP-MS corresponding to the in vitro methylation assay from panel B. Amount of arsenicals (iAs and DMA) are expressed in ppm. Error bars represents standard error of the mean (SEM) from two independent experiments (*n* = 2). ** indicates *P* < 0.01 and *** *P* < 0.001

In vitro, AS3MT catalyzes arsenic methylation within 2 h of incubation (Currier et al. 2013). We performed a time course experiment to understand the kinetics of AS3MT automethylation. In the absence of cofactors, we detected automethylation within 2 h and it reached a plateau by 8 h (Fig. 3A, lanes 2–4, and see lower exposure). The addition of cofactors immediately led to AS3MT automethylation increase up to 16 h (Fig. 3A, lanes 6–10). These observations suggest cofactors act as sources of reducing potential for both AS3MT and its automethylation. AS3MT is a cysteine-rich protein that can form intramolecular disulfide bonds (Packianathan et al. 2018). It was proposed that the reduction of these disulfide bonds by the reducing cofactors is required for regeneration of AS3MT to perform successive methylation steps (Dheeman et al. 2014). We tested whether using the reducing agent, dithiothreitol (DTT), could replace endogenous reductants and accelerate the automethylation. Remarkably, addition of an increasing amount of DTT ranging from 0.01 mM to 10 mM (Fig. S3, lanes 3–6) enhanced the AS3MT automethylation, albeit to a lesser extent than addition of the cofactors (Fig. S3, lane 2). These findings show the AS3MT automethylation preferentially occurs when the enzyme is in a reduced state.

### GSH is sufficient for AS3MT automethylation

To further decipher the reducing role of the cofactors in arsenic methylation, we assessed the effect of each individual cofactor on the automethylation of AS3MT and its methylation of arsenic. In absence of cofactors, the AS3MT automethylated signal obtained corresponds to the basal level (Fig. 3B, lane 1). Upon addition of thioredoxin reductase (TRR), thioredoxin (TRX) or NADPH, AS3MT automethylation did not increase (Fig. 3B, lanes 3–5, and see lower exposure). Addition of the GSH alone increased AS3MT automethylation to that observed by the combination of cofactors (Fig. 3B, lane 6 compared to lane 2). The faster migrating band observed in the presence of iAsIII is TRX.

To test whether the AS3MT automethylation correlates with the catalytic activity of the AS3MT, we performed an in vitro methylation assay using non-radioactive SAM followed by HG-CT-AAS analysis of As metabolites. We assayed arsenic methylation under the same conditions as optimal AS3MT automethylation (16 h). At this time point, the iAsIII methylation was complete so that little to no MAs was observed. As expected, in absence of cofactors or AS3MT, the totality of the iAs provided in the reaction remained unmethylated (iAs; Fig. 3C, controls), as previously reported (Currier et al. 2013). In the presence of all cofactors or GSH alone, DMA was observed with a proportional decrease in iAs, but not with the addition of TRR, TRX, or NADPH alone (Fig. 3C, right part of panel). Of note, AS3MT in the presence of GSH alone was less active than in the presence of all cofactors combined (Fig. 3C) as previously shown (Ding et al. 2012).

**Fig. 4:**
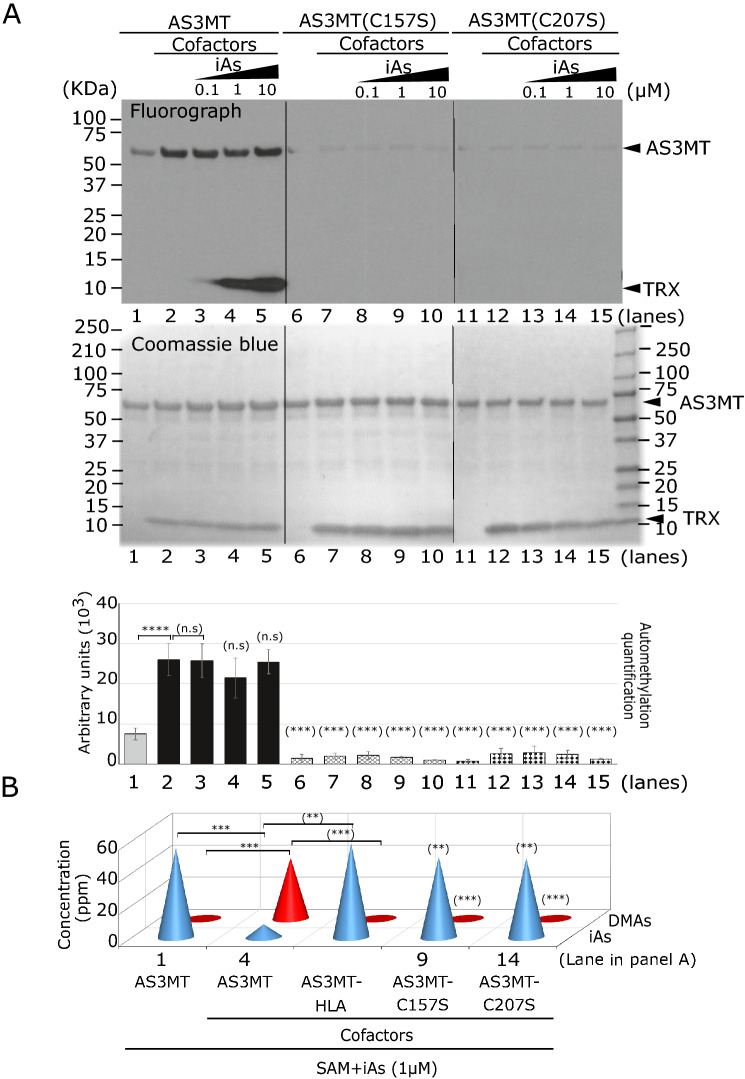
Catalytic dead AS3MT-C207S and AS3MT-C157S lack automethylation in vitro. **A** Representative in vitro methylation assay of AS3MT wild-type or cysteines mutants (C207S and C157S) in the presence of increasing concentration of iAs (0.1 µM, 1 µM and 10 µM) as indicated in the fluorograph top panel. The middle panel is the Coomassie Blue for loading control, and bottom panel corresponding to the quantification signal (arbitrary unit) obtained by fluorograph of AS3MT automethylation. Error bars represents standard error of the mean (SEM) from three independent experiments (*n* = 3). *** indicates and **** indicates *P* < 0.0001. When indicated between () is compared to lane 2. **B** Arsenic speciation profile obtained from HPLC/ICP-MS corresponding to the in vitro methylation assay from panel A as indicated. The AS3MT-HLA is used as negative control in this experiment. Amount of arsenicals (iAs, MAs and DMA) are expressed in ppm. Error bars represents standard error of the mean (SEM) from two independent experiments (*n* = 2). ** indicates *P* < 0.01 and *** indicates *P* < 0.001

In humans, iAs can bind C61, C156, and C206 located in the hAS3MT pocket, while MAs and DMA bind two and one cysteines, respectively (Li et al. 2013). These cysteines are critical to arsenic methylation (Dheeman et al. 2014). To further test whether automethylation is linked to the activity of the AS3MT, we generated two arsenic methylation dead mutants in the corresponding murine cysteine residues (AS3MT-C157S and AS3MT-C207S) as purified GST-fusion proteins and subjected them to the in vitro methylation assay. Both AS3MT-C157S and AS3MT-C207S lost their ability to self-methylate (Fig. 4A). To validate the lost catalytic capacity of these two mutants in our in vitro experiment, a Hydride Generation-CryoTrapping-Atomic Absorption Spectrometry analysis (HG-CT-AAS), was performed after 16 h. These AS3MT-C157S and -C207S, in addition to the negative control AS3MT-HLA, were unable to methylate arsenic (Fig. 4B). These findings show that C157 and C207, in addition to their known requirement for the methylation of arsenic (Thomas et al. 2007), are also required for AS3MT automethylation.

### AS3MT automethylation requires four cysteine residues: C33, C62, C157, and C207

**Fig. 5:**
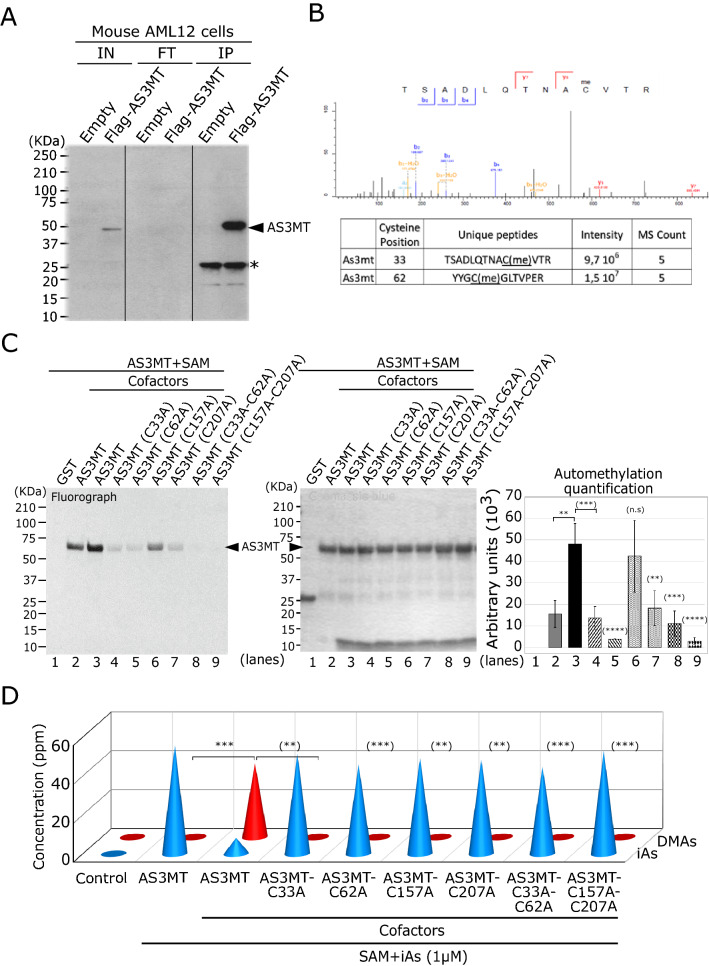
AS3MT automethylation requires cysteines C33 and C62. **A** Representative western blot of immunoprecipitation (IP (10%)) samples using flag beads in mouse AML12 cell lines expressing stably either Flag-AS3MT or empty vectors as indicated (IN = input, FT = flow through). * indicates the light chain of the anti-Flag antibody coupled to the protein A sepharose beads. **B** Top panel represents the mass spectrum (MS/MS) of methylated cysteine (AS3MTmeC33) in the identified peptides from IP in panel A. Bottom panel indicated the identified peptides containing the two identified methylated cysteine 33 and 62 from the IP-MS/MS in panel A, two independent experiments (*n* = 2) were performed. **C** Representative in vitro methylation assay (left panel), Coomassie Blue (middle panel) and corresponding quantification signal (arbitrary unit) obtained by fluorograph (right panel) of AS3MT (wild-type) and AS3MT indicated mutants (C33A, C62A, and C33A-C62A) in the presence of cofactors. Error bars represents standard error of the mean (SEM) from three independent experiments (*n* = 3). ** indicates *P* < 0.01, *** *P* < 0.001, and **** *P* < 0.0001. **D** Arsenic speciation profile obtained from HPLC/ICP-MS corresponding to the in vitro methylation assay from panel C as indicated. Amount of arsenicals (iAs and DMA) is expressed in ppm. Error bars represents standard error of the mean (SEM) from two independent experiments (*n* = 2). ** indicates *P* < 0.01 and *** *P* < 0.001

To identify the automethylated residues of mouse AS3MT in vivo, Flag-epitope tagged AS3MT was immunoprecipitated from stably transfected AML12 hepatocyte cells and the precipitates were digested with trypsin and subjected to mass spectrometry (MS/MS) for peptide detection (Fig. 5A). Our analysis reported 23 peptides covering 226 residues or ~ 60% of AS3MT (Fig. S4). The MS/MS spectrum revealed methylated C33 and C62 in a total of four cysteines detected in our coverage (Fig. 5B). The other two detected cysteines, C87 and C252, were not methylated, while cysteines forming the active site, C157 and C207, were not detected in our assay, even with various proteinase digestion methods. This approach showed that AS3MT is methylated in cells and defined C33 and C62, as automethylation sites.

To further characterize the cysteines responsible for automethylation and the methylation of arsenic, C33 and C62 were converted to a neutral alanine in single mutants C33A and C62A or a dual mutant C33A-C62A, and then assessed for in vitro methylation assays. Both AS3MT-C33A and AS3MT-C62A showed a dramatic decrease of automethylation (Fig. 5C, lanes 4 and 5). Furthermore, AS3MT-C33A-C62A completely abrogated the ability to automethylate, suggesting C33 and C62 represent the two sites of automethylation. Using HG-CT-AAS, AS3MT-C33A, AS3MT-C62A and AS3MT-C33A-C62A were all defective in their ability to methylate iAs in the presence of cofactors (Fig. 5D). These findings show that AS3MT automethylation is governed by at least four cysteines C33, C62, C157, and C207, which are the same residues needed to catalyze arsenic methylation.

### AS3MT automethylation does not prevent iAs methylation

**Fig. 6:**
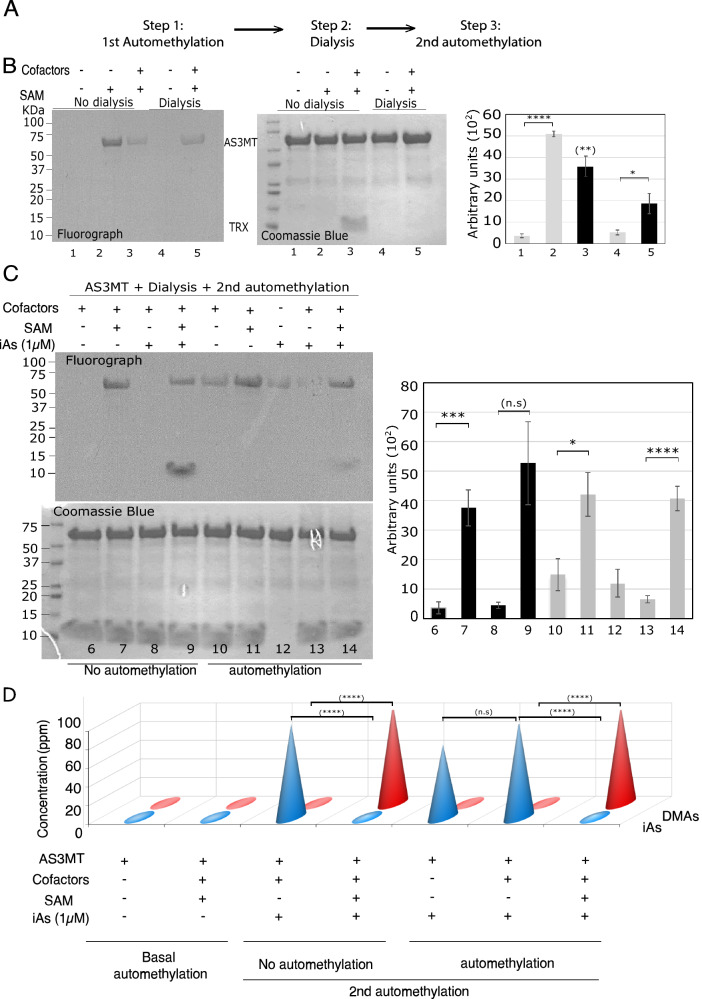
Automethylated AS3MT can methylate iAsIII.** A** Experimental design to test 2nd methylation step. **B** Representative fluorograph (left panel) and Coomassie Blue (right panel) show that automethylated AS3MT is retained, and cofactors are removed following dialysis. **C** Purified AS3MT, non-automethylated and automethylated, were used in 2nd in vitro methylation reactions with and without cofactors, [H^3^] SAM, and/or 1 µM iAsIII. Representative fluorograph (left panel) and Coomassie Blue (right panel) are presented. **D** Arsenic speciation profile obtained from HPLC/ ICP-MS corresponding to the in vitro methylation assay from panel C where iAsIII was added. Amount of arsenicals (iAs and DMA) are expressed in ppm. Error bars represents standard error of the mean (SEM) from three independent experiments (*n* = 3). * indicates *P* < 0.05, ** indicates *P* < 0.01, *** *P* < 0.001, and **** *P* < 0.0001

We next asked whether prior automethylation influenced iAs methylation. We purified GST-AS3MT, both unmethylated (no automethylation) and automethylated with SAM with cofactors (automethylation). These protein preparations were dialyzed with a 20 K cutoff to remove cofactors (Fig. 6B, lanes 3 and 5) and SAM. These preparations were used in subsequent methylation reactions to methylate iAs. The ‘no automethylation’ GST-AS3MT behaved like GST-AS3MT as it automethylated with cofactors and catalyzed the conversion of iAs to DMA (Fig. 6C lane 9, and 6D). However, automethylated GST-AS3MT did not methylate iAs in the absence of exogenous SAM (Fig. 6D, lane 5), suggesting automethylated AS3MT does not act as an intermediate step in iAs methylation. The ‘automethylated’ GST-AS3MT levels increased with [^3^H] SAM with and without cofactors (Fig. 6C, lane 11 and 14 versus lane 10). Both enzymes were equally able to convert iAs to DMA in the presence of SAM (Fig. 6D). These findings suggest that the automethylation of AS3MT is consistent with an active AS3MT capable of methylating iAs.

## Discussion

**Fig. 7:**
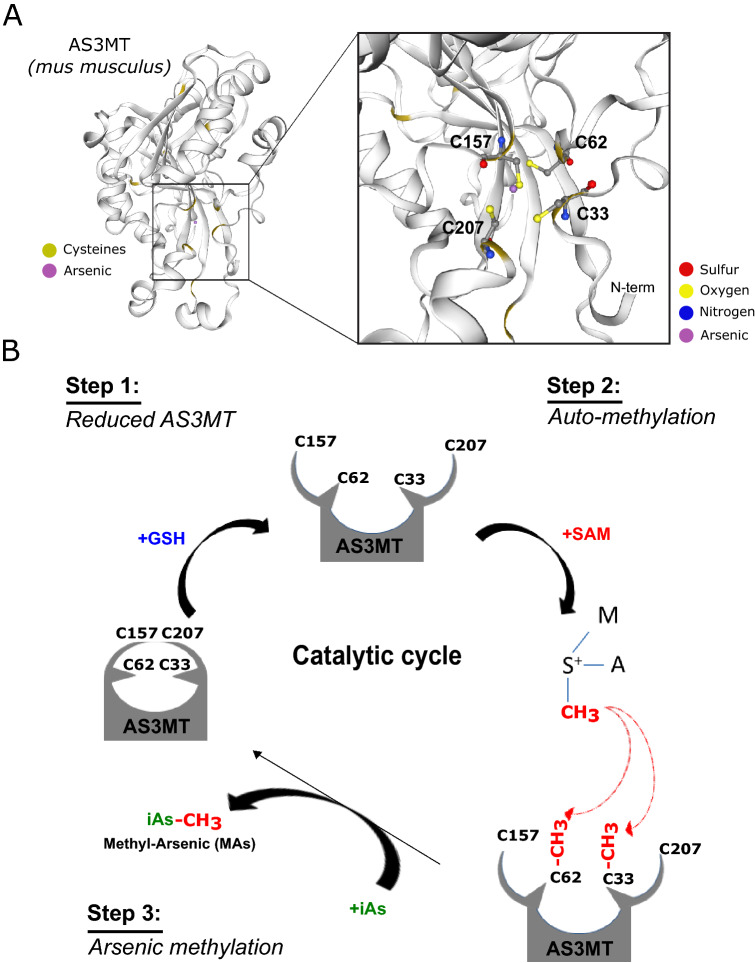
Proposed model for the mechanism of AS3MT-catalyzed methylation. **A** Murine AS3MT model (generated using Swiss Model https://swissmodel.expasy.org) showing the entire protein and the binding pocket (inset). On the left, cysteines are colored yellow, and arsenic is in purple. On the right, yellow is sulfur, red is oxygen, blue is nitrogen, and purple is arsenic. **B** The enzymatic methylation of arsenic occurs on the AS3MT enzyme in three steps: Step1: GSH reduced the disulfide bond in the catalytic pocket involving four cysteines C33, C62, C157 and C207 (herein, we show one disulfide bond between C157-C207), which results in the opening of the catalytic pocket. Step2: When these cysteines are released, one methyl group can be accepted by one cysteine or multiple cysteines. Step3: One methyl group is transferred from the catalytic pocket onto the arsenic iAs to accomplish the methyltransferase activity

Inorganic arsenic (iAs) is metabolized in a series of methylation steps catalyzed by AS3MT forming MAsIII, DMAIII and the volatile trimethylarsine (TMA). This process is conserved across species from bacteria to man. The methylation of arsenic is coordinated by three conserved cysteine residues proposed to participate in catalysis, namely C62, C157, and C207 in mouse AS3MT (Fig. 7). The current model of iAs methylation requires a series of intramolecular disulfide bonds to form before the enzymatic methylation of arsenite (iAs3+) (Dheeman et al. 2014). In the presence of endogenous reductants, these disulfide bonds are reduced, leading to the methylation of the iAs in the presence of the methyl group donor SAM. Using in vitro methylation assays, we find that AS3MT undergoes an automethylation step in the absence of iAs at C33 and C62. This automethylation was enhanced by its cofactor GSH or even DTT, suggesting that reduced cysteines accept methyl groups from SAM (Fig. 7). Following the addition of iAs, the automethylation of AS3MT is decreased, as transfer of these methyl groups completes the first round of iAs methylation. Furthermore, using a Flag-AS3MT immunoprecipitation coupled to MS/MS, we identified both C33 and C62 as acceptors of methyl groups in vivo. Site-directed mutagenesis (C to A) revealed that three of the previously described cysteines were required for AS3MT automethylation step. Our results identify a novel discovery of the automethylation of AS3MT and adds a new feature of its enzymology.

Automodification occurs frequently in post-translational modifying enzymes and serves to regulate activity and specificity of the enzyme. This modulation is well characterized for protein kinases where autophosphorylation of the activation loop causes a conformational change for substrate accessibility (Pawson 2002). In the methylome kingdom, many methyltransferases are automodified at the same type of residues as their substrates (Clarke 2013). However, the functional consequences of these automethylation events, for the most part, remain unknown. Arginine methyltransferases PRMT6 (Frankel et al. 2002), PRMT7 (Geng et al. 2017) and PRMT8 (Sayegh et al. 2007) automethylate. Automethylation of PRMT8 occurs in the absence of endogenous substrates, decreasing the affinity of PRMT8 for SAM, and therefore, is a way to downregulate the enzyme in the absence of substrate (Sayegh et al. 2007). Lysine methyltransferase MLL1 (Patel et al. 2014), SUV39H2 (Iglesias et al. 2018; Piao et al. 2016), PRDM9 (Koh-Stenta et al. 2017), and G9a (Chin et al. 2007) also automethylate. In the case of G9a, automethylation creates a new binding site for the methyl-lysine interactor HP1 (Chin et al. 2007). Akin to protein kinases, automethylation of an internal loop in Clr4 (Suv39h) promotes a conformational switch to enhance the activity of Clr4 (Iglesias et al. 2018) and automethylation of the PRC2 also modulates its histone methyltransferase activity (Lee et al. 2019; Wang et al. 2019).

Cysteine methylation generates the chemically stable S-methylcysteine, somewhat resembling methionine (Clarke 2013). Notably, cysteines are frequently present in active sites where they function as strong nucleophiles and their methylation may sterically block their nucleophilic abilities. Interestingly, methionine in the active site of LaeA was shown to be methylated generating a S-methylmethionine (Patananan et al. 2013). Thus, it is possible that the active site cysteine may be able to receive two methyl groups generating S-dimethylcysteine, a reactive sulfonium, able to transfer a methyl group to an exogenous substrate or a neighboring residue. However, our data do not support the possibility of a S-dimethylcysteine, a reactive sulfonium as an intermediate to methylate iAs. This is based on the remethylation experiment in the absence of added SAM (Fig. 6C, D). One can argue that the stoichiometry of methylation in Fig. 6 is not high enough to observe such a phenomenon. We concluded that the S-methylcysteines are present when AS3MT is active and whether it plays a role in activating and in methylation reaction of iAs remains to be shown.

## Supplementary Information

Below is the link to the electronic supplementary material.

Supplementary file1 (PDF 70 KB)

## Data Availability

All data are contained with the article.
