# Supplementary material for: Arsenic 3 methyltransferase (AS3MT) automethylates on cysteine residues in vitro
Source: Arch Toxicol. 2022 Mar 4;96(5):1371–86. doi: 10.1007/s00204-022-03248-8 (PMC9013690; doi:10.1007/s00204-022-03248-8)
Supplement: Supplementary file 1 — Supplementary file1 (PDF 70 KB) [file 204_2022_3248_MOESM1_ESM.pdf]

Supplementary Figure Legends

Figure S1. AS3MT does not methylate histones. *In vitro* methylation assay (left panel) and Coomassie Blue stained gel (right panel) of GST-AS3MT and histones H2A, H2B, H3, or H4, in presence of indicated factors (0.4 μM SAM and cofactors: 0.2 μM TRR, 10 μM TRX, 300 μM NADPH, 1 mM GSH) after a 16 hr incubation at 37°C.

Figure S2: Both mouse and human AS3MT automethylate. *In vitro* methylation assay (left panel) and Coomassie Blue stained gel (right panel) of GST-murine AS3MT or MBP-human AS3MT, in presence of 0.4 μM SAM and cofactors: 0.2 μM TRR, 10 μM TRX, 300 μM NADPH, 1 mM GSH after a 16 hr incubation at 37°C.

Figure S3: Addition of DTT enhances automethylation of AS3MT. *In vitro* methylation assay (top panel) and Coomassie Blue stained gel (bottom panel) of GST-AS3MT in presence of 0.4 μM SAM and cofactors: 0.2 μM TRR, 10 μM TRX, 300 μM NADPH, 1 mM GSH (lane 2) or increasing concentration of DTT after a 16 hr incubation at 37°C. Quantification of two independent experiments is shown (n=2), error bars represents standard error of the mean (SEM).

Figure S4: Sequence coverage of AS3MT from mass spectrometry. Yellow highlight indicates residues identified by MS/MS. Blue are the cysteine residues identified by MS/MS as automethylated, while red of those cysteines shown to be important in automethylation, but not covered in MS/MS experiments.
